# Aromatase-Inhibitor-Induced Musculoskeletal Inflammation Is Observed Independent of Oophorectomy in a Novel Mouse Model

**DOI:** 10.3390/ph15121578

**Published:** 2022-12-17

**Authors:** Nicholas A. Young, Jeffrey Hampton, Juhi Sharma, Kyle Jablonski, Courtney DeVries, Anna Bratasz, Lai-Chu Wu, Maryam Lustberg, Raquel Reinbolt, Wael N. Jarjour

**Affiliations:** 1 Department of Internal Medicine, Division of Rheumatology and Immunology, The Ohio State University Wexner Medical Center, Columbus, OH 43210, USA; 2 Department of Medicine, WVU Cancer Institute, WVU Rockefeller Neuroscience Institute, West Virginia University, Morgantown, WV 26506, USA; 3 Small Animal Imaging Core, The Ohio State University Wexner Medical Center, Columbus, OH 43210, USA; 4 Department of Biological Chemistry and Pharmacology, The Ohio State University Wexner Medical Center, Columbus, OH 43210, USA; 5 Smilow Cancer Hospital/Yale Cancer Center, New Haven, CT 06519, USA; 6 Department of Internal Medicine, The James Cancer Hospital, The Ohio State University Wexner Medical Center, Columbus, OH 43210, USA

**Keywords:** breast cancer, hormone-receptor-positive cancer, estrogen, aromatase inhibitor, aromatase-inhibitor-induced arthralgia (AIIA)

## Abstract

Aromatase Inhibitors (AIs) block estrogen production and improve survival in patients with hormone-receptor-positive breast cancer. However, half of patients develop aromatase-inhibitor-induced arthralgia (AIIA), which is characterized by inflammation of the joints and the surrounding musculoskeletal tissue. To create a platform for future interventional strategies, our objective was to characterize a novel animal model of AIIA. Female BALB/C-Tg(NFκB-RE-luc)-Xen mice, which have a firefly luciferase NFκB reporter gene, were oophorectomized and treated with an AI (letrozole). Bioluminescent imaging showed significantly enhanced NFκB activation with AI treatment in the hind limbs. Moreover, an analysis of the knee joints and legs via MRI showed enhanced signal detection in the joint space and the surrounding tissue. Surprisingly, the responses observed with AI treatment were independent of oophorectomy, indicating that inflammation is not mediated by physiological estrogen levels. Histopathological and pro-inflammatory cytokine analyses further demonstrated the same trend, as tenosynovitis and musculoskeletal infiltrates were detected in all mice receiving AI, and serum cytokines were significantly upregulated. Human PBMCs treated with letrozole/estrogen combinations did not demonstrate an AI-specific gene expression pattern, suggesting AIIA-mediated pathogenesis through other cell types. Collectively, these data identify an AI-induced stimulation of disease pathology and suggest that AIIA pathogenesis may not be mediated by estrogen deficiency, as previously hypothesized.

## 1. Introduction

Aromatase inhibitors (AIs) block physiological estrogen production in peripheral tissues and significantly improve overall survival rates by reducing disease recurrences in patients with hormone-receptor-positive breast cancer [1]. AI-induced arthralgias (AIIAs) are experienced by approximately 35–50% of women taking AIs and are characterized by joint stiffness, pain, tissue inflammation, and swelling [2,3,4]. These adverse events can significantly impact patient quality of life and contribute to early discontinuation and/or non-adherence to therapy [5], leading to termination in 10–20% of patients [3]. Moreover, recent reports suggest that poorer patient outcomes occur as a result of non-adherence/non-compliance with AI therapy [6], and data indicate that AI treatment extension would offer more protection from tumor relapse. In summary, AIIAs are a significant clinical obstacle to breast cancer treatment with AIs in the maintenance phase because of the high incidence of AIIA occurrence and the negative impact on outcomes when treatment is suspended.

The diagnosis of AIIA is generally clinical, lacking standardized diagnostic criteria and confirmatory testing. Furthermore, there are no defined methods to objectively measure the effect of interventions designed to prevent or minimize toxicity. While the exact pathogenic mechanisms of AIIA remain to be definitively elucidated [4], estrogen deprivation directly mediated by AIs has been hypothesized, and some preclinical studies have demonstrated estrogen to have a protective effect in arthritis and on the expression of pro-inflammatory genes [7,8,9]. Moreover, there is evidence that the pro-inflammatory cytokines IL-1, IL-6, and TNF-α are all increased in the first several years following menopause, which coincides with lower estrogen levels and a period during which joint symptoms are prevalent [10]. Other studies have postulated that estrogen may modulate spinal opioid anti-nociceptive activity to have a pain-modulating effect [11]. In addition, the upregulation of inflammatory pathways and pro-inflammatory cytokines may be regulated by estrogen [12], especially in female-predominant autoimmune diseases (reviewed in [13]). Collectively, a better understanding of the underlying molecular and pathogenic mechanisms of AIIA development and the role of estrogen specifically in this process may enhance the efficacy of interventions aiming to prevent or treat this condition.

**Figure 1:**
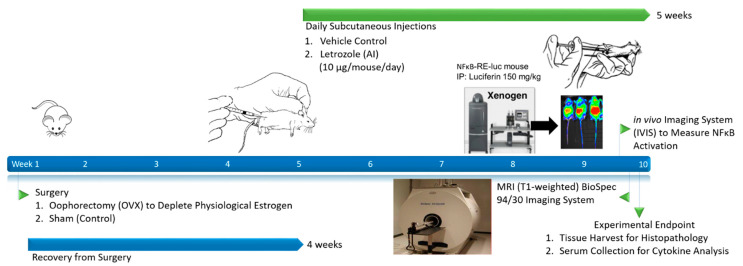
Schematic of experimental outline and sample collection establishing a novel murine model of AIIA.

Translational animal models are a powerful tool to study the pathophysiology of human disease and to evaluate putative medical countermeasures. Although mouse models have been frequently used to study inflammation associated with rheumatologic disease, there are few well-developed preclinical models that could potentially be used to study the pathophysiology of AIIAs. Letrozole is the most potent oral AI used in patients with breast cancer [8], and it was used in this study to determine whether inflammation can indeed be detected following treatment. In order to recapitulate a post-menopausal state, an oophorectomy procedure using previously established animal models was performed on female mice [14]. This well-established model demonstrated more than a 30% reduction in systemic estrogen levels approximately two weeks after the procedure [15]. Following daily treatment with letrozole, our results demonstrate the successful detection of AI-induced inflammatory responses and establish a novel animal model of AIIA (Figure 1; Graphical Abstract). Interestingly, AI-induced inflammation was observed irrespective of the oophorectomy procedure, which indicates that AIIA pathogenesis is independent of systemic physiological estrogen levels.

## 2. Results

### 2.1. Letrozole-Induced Inflammation Detection via MRI and IVIS in Murine Hind Limbs

**Figure 2:**
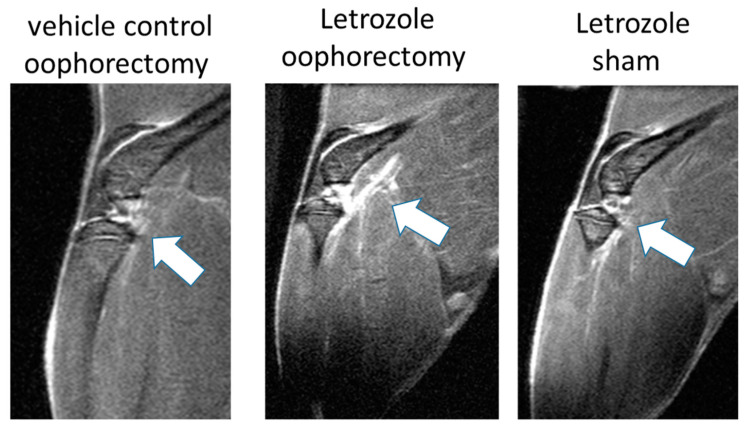
Enhanced MRI signals detected in the legs of mice receiving aromatase inhibitor treatment. Female NFkB-RE-luc mice were injected subcutaneously with the aromatase inhibitor letrozole (10µg/mouse/day) or vehicle control (0.3% hydroxypropyl cellulose) following oophorectomy or control surgery (sham). MRIs were conducted on live mice after 5 weeks of treatment. Enhanced signals (white arrows) were observed in knees and surrounding muscle tissue via MRI (T1-weighted) with letrozole treatment, and these responses were detected in NFkB-RE-luc mice irrespective of the oophorectomy procedure.

After 5 weeks of daily subcutaneous letrozole injections, the knee joints and surrounding hind limb tissues of mice were imaged on a BioSpec 94/30 micro-MRI with gadolinium enhancement under anesthesia. The analysis of the knee joints and proximal anatomy via the MRI showed enhanced signal detection in the joint space and surrounding tissue following letrozole treatment in oophorectomized NFκB-RE-luc mice (Figure 2). Surprisingly, enhanced MRI detection was also demonstrated in non-oophorectomized (sham surgery) mice that were treated with letrozole. Although the MRI signal detection was lower without the oophorectomy procedure, letrozole did elevate responses relative to those of the untreated controls (daily vehicle injections following an oophorectomy procedure), which indicates that AIIA pathophysiology may be independent of the physiological levels of estrogen.

**Figure 3:**
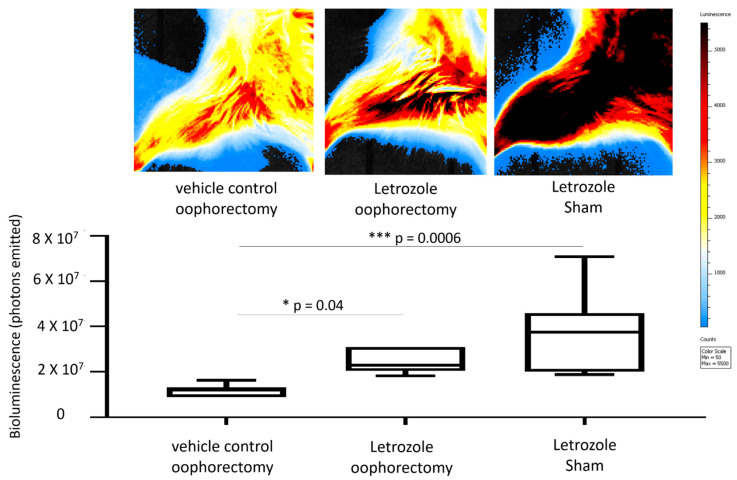
Bioluminescent imaging of NFκB activity shows aromatase-inhibitor-induced upregulation with and without oophorectomy. Female NFkB-RE-luc mice were treated with letrozole following an oophorectomy procedure. Controls included sham surgery and vehicle control treatment (0.3% hydroxypropylcellulose; HPC). NFκB activity was measured on the in vivo imaging system (IVIS) approximately at the 5-week timepoint. IVIS (top) and quantitation of bioluminescent signals (bottom) demonstrate induction of NFκB activity both with and without oophorectomy relative to control mice. Values are the mean ± SEM with indicated *p* values calculated via one-way ANOVA with Tukey’s corrections for multiple comparisons. * *p* ≤ 0.05; *** *p* ≤ 0.001. All values of *p* ≤ 0.05 were considered statistically significant.

The transcription factor NFκB serves as a pivotal mediator of inflammatory responses. In order to measure NFκB activation that might be associated with aromatase inhibitor treatment, bioluminescent imaging was performed on the NFκB-RE-luc mice after 5 weeks of letrozole treatment using luciferin and an in vivo imaging system (IVIS). The digital quantitation of emitted photons from the hind limbs showed significantly enhanced NFκB activation with letrozole treatment compared to oophorectomized controls receiving vehicle injections (Figure 3). Specifically, letrozole-mediated NFκB activation increased by 45% (*p* = 0.04) in the mice with an oophorectomy and by 65% (*p* = 0.0006) in the mice with sham surgeries. In concordance with the MRI data above, the activation of NFκB was observed with and without oophorectomy, which suggests that the induction of letrozole-induced inflammatory responses is not dependent on estrogen levels.

### 2.2. Histopathological Analysis Shows That Letrozole Increases Macrophage Infiltration

**Figure 4:**
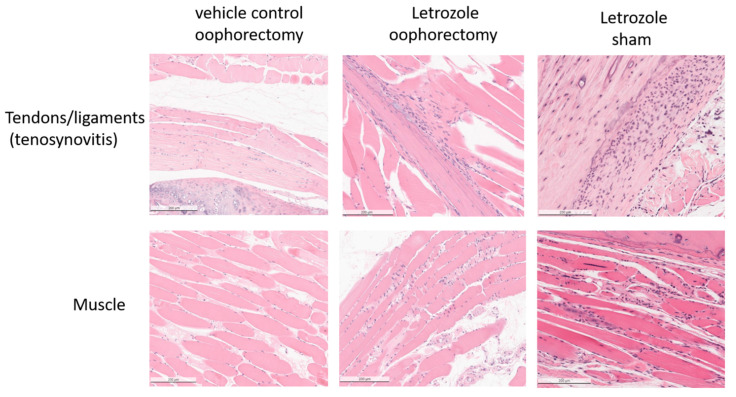
Histopathological analysis of mice receiving aromatase inhibitor treatment indicates tenosynovitis and myositis. H&E staining of tissue sections taken from female NFκB-RE-luc mice after 5 weeks of subcutaneous letrozole treatment. Muscle tissue sections were dissected from legs and processed for H&E staining and histopathology. Cellular infiltrates are present in tendons and muscles with treatment of the aromatase inhibitor letrozole independent of oophorectomy or sham surgery.

**Figure 5:**
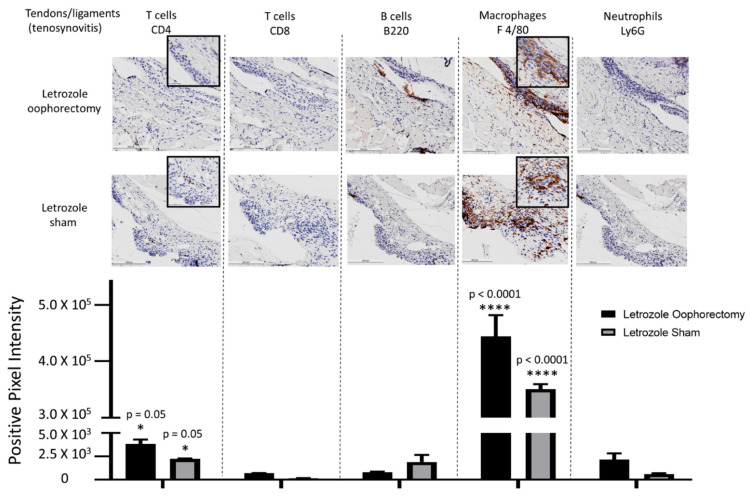
IHC for immune cell subtypes in tenosynovitis infiltrates shows a macrophage-mediated inflammatory response induced by aromatase inhibitor treatment. Following oophorectomy or control (sham) surgery and induction of AIIA with daily letrozole injections, mouse legs were collected and processed for histopathological assessment as described in the Materials and Methods Section. To evaluate immune cell subtypes in the tendons, IHC staining of CD4+ T cells, CD8+ T cells, B220+ B cells, F 4/80+ macrophages, and Ly6G+ neutrophils was performed. Data were analyzed via two-way ANOVA for group-wise comparisons relative to each IHC stain. Values are the mean ± SEM, with indicated *p* values determined following Tukey’s corrections for multiple comparisons to measure the statistical change between IHC cell stain/subtype. * *p* ≤ 0.05; **** *p* ≤ 0.0001. All values of *p* ≤ 0.05 were considered statistically significant compared to all other stains individually.

**Figure 6:**
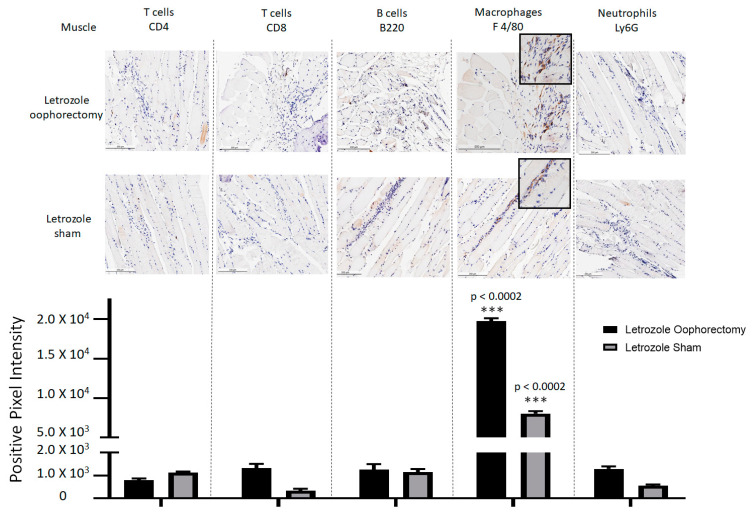
Macrophage-predominant inflammation of muscle tissue detected with AIIA induced by aromatase inhibitor treatment. As outlined in the Materials and Methods Section, AIIA was induced in mice by administration of daily letrozole injections subsequent to oophorectomy or control (sham) surgery. Histopathology of muscle tissue was evaluated via IHC staining of CD4+ T cells, CD8+ T cells, B220+ B cells, F 4/80+ macrophages, and Ly6G+ neutrophils to characterize immune cell subtypes. Data analysis was performed using two-way ANOVA for group-wise comparisons relative to each IHC stain. Two-way ANOVA with Tukey’s correction for multiple comparisons was performed to evaluate the statistical changes between IHC cell stain/subtype. Values shown are the mean ± SEM, with indicated *p* values relative to other immune cell subtypes. *** *p* ≤ 0.001.All values of *p* ≤ 0.05 were considered statistically significant relative to each stain individually.

At the experimental endpoint, the hind limbs of the mice were collected for histopathological analysis. H&E staining of tissue sections demonstrated mild tenosynovitis and inflammatory muscle tissue infiltrates (Figure 4) in both groups of mice treated with letrozole with and without oophorectomy. To characterize the immune cell subtypes present in these infiltrates, IHC was performed to detect the CD4+ T cells, CD8+ T cells, B220+ B cells, F 4/80+ macrophages, and Ly6G+ neutrophils present in the tendons and ligaments (Figure 5), as well as of other musculoskeletal tissue (Figure 6). The results show that the infiltrates in these tissues consisted largely of macrophages. Digital image analysis via two-way ANOVA was used to estimate how the mean quantitative values changed according to the levels of the categorical variables. Here, the categories that were compared statistically were immune cell subtypes (i.e., IHC staining categories). The data demonstrate that macrophage detection relative to the other immune cell subtypes was greatest in areas of tenosynovitis (*p* < 0.0001), but also significantly elevated in muscle tissue (*p* < 0.0002). Additionally, CD4+ T cells were also detected to a lesser extent in the histopathological analysis of the tendons/ligaments and muscle tissue; the digital quantification of CD4 IHC was statistically significant in tenosynovitis infiltrates with letrozole stimulation when compared to the other immune cell subtypes (*p* = 0.05).

### 2.3. AIIA Mouse Model Shows Induction of Pro-Inflammatory Cytokines

**Figure 7:**
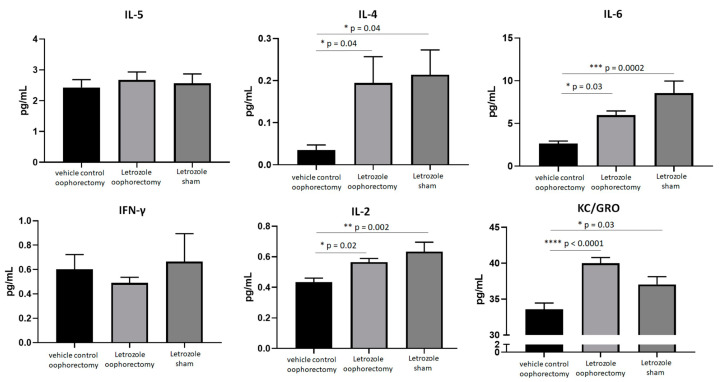
Aromatase inhibitors induce pro-inflammatory cytokine levels in the serum of NFκB-RE-luc mice independent of physiological estrogen depletion. Female NFκB-RE-luc mice underwent oophorectomy or control (sham) surgery and were treated with the aromatase inhibitor letrozole for comparison to negative (vehicle) controls. Cytokine levels from serum were measured via electrochemiluminescence detection using a pro-inflammatory panel. Values are the mean ± SEM, with indicated *p* values calculated from one-way ANOVA with Tukey’s corrections for multiple comparisons. * *p* ≤ 0.05; ** *p* ≤ 0.01; *** *p* ≤ 0.001; **** *p* ≤ 0.0001. All values of *p* ≤ 0.05 were considered statistically significant.

To measure systemic inflammation biomarkers, serum was prepared from mice at the experimental endpoint and analyzed by using an electrochemiluminescent ELISA. The pro-inflammatory cytokine levels of IL-2, IL-4, IL-6, and KC/GRO (CXCL1) were significantly elevated in the mice with letrozole treatment when compared to the untreated, oophorectomized control mice irrespective of the oophorectomy procedure (Figure 7). Relative to the oophorectomized mice receiving vehicle control injections, letrozole induced systemic IL-4 (*p* = 0.04), IL-6 (*p* = 0.03), IL-2 (*p* = 0.02), and KC/GRO (CXCL1) (*p* < 0.0001) following the oophorectomy procedure. Despite a lack of physiological estrogen depletion following sham surgery, letrozole significantly enhanced the serum expressions of IL-4 (*p* = 0.04), IL-6 (*p* = 0.0002), IL-2 (*p* = 0.002), and KC/GRO (CXCL1) (*p* < 0.03). These data further support the hypothesis that letrozole induces a pro-inflammatory response in this murine model of AIIA independent of estrogen levels.

### 2.4. Letrozole Treatment of Human PBMCs In Vitro Does Not Produce an Observable Genomic Effect

**Figure 8:**
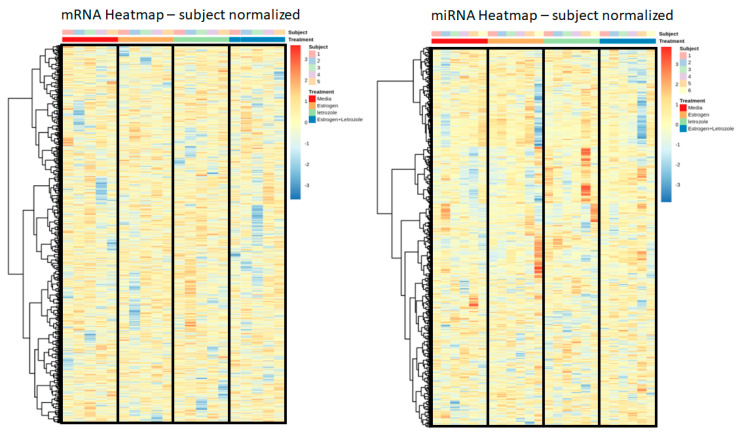
Genome-wide expression analysis of human PBMCs does not demonstrate an aromatase-inhibitor-mediated response. Freshly isolated human PBMCs were stimulated in vitro with estrogen, letrozole, or a combination of both for a comparison relative to untreated control samples. RNA sequencing was preformed to evaluate overall expression levels of mRNA and miRNA targets with each treatment sample. Data from each subject were normalized to control values, and relative expression levels are presented as heatmaps to evaluate genome-wide treatment effects on mRNA (**left**) and miRNA (**right**) transcripts.

To explore the genomic influence of letrozole on human PBMCs and to provide insight into the molecular mechanisms of inflammation in AIIA, healthy human PBMCs from five pre-menopausal females were isolated and then stimulated under four conditions: (i) untreated, (ii) estrogen, (iii) letrozole, and (iv) estrogen + letrozole. Cell pellets were collected at 36 hrs, and RNA sequencing (RNAseq) for mRNA and (micro) miRNA transcriptional targets was performed. Targets with low counts were removed via edgeR::filterByExpr, which uses embedded filtering for sequencing depth and experimental design considerations. After filtering, 24,511 genomic targets were included in the mRNA analysis, and 1599 were included in the miRNA analysis: these correlate to 85% and 92% of the total, respectively. Normalization was carried out using the trimmed mean of M-values (TMM) method and indicated that the filtering was adequate for a downstream comparative evaluation. Using these data, the results were normalized to untreated values for each patient individually in order to account for patient-to-patient variability in the datasets. To evaluate the genome-wide effects of these treatments, heatmaps were created for the mRNA and miRNA targets with hierarchical clustering using Pearson’s correlation distance measure and Ward’s clustering criterion with squared dissimilarities. The rows were scaled so that sample locations could be compared across a gene, and the number of genes was reduced to >10,000 for mRNA by further filtering the genes that had the lowest standard deviations. The results for both mRNA and miRNA did not demonstrate any strong or biologically meaningful clustering (Figure 8), which indicates that the induction of AIIA is unlikely mediated by a direct effect on the regulation of PBMC gene expression.

## 3. Discussion

AIs, including letrozole, are approved prophylactic, long-term treatments for patients with hormone-receptor-positive breast cancer [1]. Although this adjuvant treatment strategy has demonstrated efficacy in reducing disease relapse via the suppression of physiological estrogen levels, one complicating immune-related adverse event is AIIA. AIIA is clinically characterized by joint pain, tenosynovitis, and musculoskeletal inflammation [2,3,4], with the proposed pathogenesis resulting presumably from estrogen deprivation [9,10,11]. Unfortunately, approximately half of patients receiving AI therapy develop AIIA, which results in treatment discontinuation in up to 20% of this cohort [3]. Since the suspension of AI therapy is associated with an increased risk of relapse [6], the prevention or proactive management of AIIA could lead to better patient outcomes via prolonged adherence to treatment. With early breast cancer detection leading to better overall patient outcomes and AI treatment recommendations extending from five to ten years [7], more patients will inevitably develop AIIA. To establish an animal model of AIIA, we treated mice with letrozole and studied the responses both with and without oophorectomy. The results from this study establish a novel animal model of AIIA that can be leveraged in future work to evaluate novel interventions and diagnostics to prevent or ameliorate AIIAs, which may ultimately translate into improved clinical outcomes.

Our results demonstrate macrophage-predominated infiltrates in both the tendons and surrounding musculoskeletal tissue and significantly induced levels of IL-4, IL-2, and IL-6, as well as chemokine KC/GRO (CXCL1), with daily letrozole injections. To the best of our knowledge, this is the first study to characterize AIIA-associated infiltrates histopathologically; therefore, these data may help inform which putative AIIA prophylaxis options to evaluate in future studies. Since the inflammatory response consists mostly of macrophages, treatments directed at this specific immune cell subtype should be prioritized. Accordingly, in our previous studies evaluating the anti-inflammatory effects of nano-emulsified curcumin, we demonstrated a reduction in NFκB activation, macrophage-specific chemoattractant MCP-1 secretion, in vitro macrophage migration using both primary cells and cell lines, and macrophage recruitment in a mouse model of peritonitis [16]. Similar to our strategy used in curcumin-containing lipid nanoparticles [16], therapeutic strategies leveraging selective macrophage phagocytosis of lipid particles loaded with immunomodulatory biocargo should also be considered [17]. In addition to these potential interventions, the use of disease-modifying antirheumatic drugs (DMARDs) that have demonstrated the capacity to drive macrophage polarization from the pro-inflammatory M1 state to the wound-healing M2 state may also be effective treatments for AIIA [18]. Specifically, Abatacept (a small molecule inhibitor that blocks T-cell activation by binding to CD80 or CD86 extracellularly in order to prevent interaction with co-stimulatory receptor CD28), Etanercept (a soluble TNF receptor that competitively binds TNF-α and TNF-β), Infliximab (a mAb that binds to TNF-α), Rituximab (a mAb that binds to CD20 and inhibits B-cell activation), and Tocilizumab (an anti-IL-6 mAb) are all DMARDs to consider in future studies evaluating the efficacy of preventing or treating AIIA. The detection of enhanced systemic pro-inflammatory cytokine/chemokine expression with letrozole treatment indicates that these pro-inflammatory mediators may stimulate the observed macrophage-mediated inflammatory responses. Considering that IL-2 and IL-6 are well-characterized pro-inflammatory Th1-mediated cytokines [19] and that KC/GRO (CXCL1) is associated with M1 polarization [20], these immunomodulators could also be selectively targeted to suppress AIIA pathogenesis. While the overexpression of the Th2/M2-associated, wound-healing cytokine IL-4 may be a compensatory mechanism that has been previously observed in inflammatory disease [21], this hypothesis will require additional investigation to elucidate more definitively. Collectively, the results from this study indicate that macrophage-mediated therapeutic targets associated with M1 polarization and inflammatory disease pathogenesis should be prioritized in future strategies to ameliorate AIIA.

The BALB/C-Tg(NFκB-RE-luc)-Xen mouse carries a transgene containing 6 NFκB-responsive elements from the immediate early CMVα promoter placed upstream of a basal SV40 promoter and a modified firefly luciferase cDNA [22]. While the enhancement of bioluminescent signal detection observed in this model is a direct consequence of NFκB activation of regulatory elements resulting in luciferase expression in immune system cells during an inflammatory response [23,24], the etiologic agent responsible for this response does not have to directly activate NFκB. In this study, we injected NFκB-RE-luc mice with letrozole following recovery from an oophorectomy procedure, and we measured NFκB activity via bioluminescent imaging locally in the tibiofemoral region. While letrozole treatment did result in both tenosynovitis and musculoskeletal inflammation independent of physiological estrogen levels, the in vitro treatment of human PBMCs did not result in a measurable effect over gene expression. Consequently, these results suggest that the pathogenic mechanism of letrozole-induced gene responses may be derived from cell types other than PBMCs. In concordance, the interaction of synovial fibroblasts with tissue-resident macrophages has been shown to be associated with synovitis in autoimmune disease pathogenesis [25]. In this model of rheumatoid arthritis, the fibroblast secretion of IL-6, CXCL12, and CCL2 in the synovial microenvironment, as well as the expression of an IFN-γ gene signature, stimulates the macrophage-mediated inflammatory response. Moreover, chondrocytes have a well-established role in inflammatory disease pathogenesis of arthralgias and should also be considered in future in vivo studies evaluating the mechanism of AIIA induction. Chondrocytes can secrete IL-1β into the synovium to stimulate NFκB expression in immune cells and MMP-13, which degrades the extracellular matrix in the synovial compartment [26]. Consequently, the activation of macrophages in the synovium by chondrocytes and/or synovial fibroblasts may be the impetus of a broader inflammatory response ultimately leading to tenosynovitis and musculoskeletal inflammation. Future studies using our murine model will characterize the roles of both chondrocytes and synovial fibroblasts, as well as tissue-resident macrophages, in letrozole-induced inflammatory responses.

Interestingly, our data indicate that letrozole-induced inflammatory responses occur independent of physiological estrogen levels, as mice both with and without oophorectomy produced similar results in this study. This is particularly important because the current prevailing hypotheses speculate that AIIA pathogenesis is mediated by estrogen suppression [9,10,11]. However, while AI treatment does suppress physiological estrogen, systemic reduction is not consistent from patient-to-patient and is largely inconsequential in patients with clinical obesity and extended treatment [27]. These data suggest that non-obese patients with statistically lower levels of estrogen following AI treatment should have a higher incidence of AIIA. On the contrary, patients with obesity are actually more likely to develop AIIA than their non-obese counterparts, indicating that the AI-mediated induction of AIIA may indeed be estrogen-independent [28]. Similarly, estrogen replacement therapy has been shown to be efficacious in post-menopausal women with arthralgia not resulting from joint replacement due to hip fracture, according to the Women’s Health Initiative [29]. Considering these observations from human studies along with the results from our experiments, the prevailing estrogen-dependent hypothesis explaining the pathogenesis of AIIA should be further investigated and reconsidered in the context of AI-mediated effects independently.

These results establish a novel mouse model of AIIA; thus, our future studies with this model will be directed towards the further characterization of this inflammatory mechanism to provide insight into potential therapeutic strategies directed at mitigating this adverse inflammatory burden. Additionally, a longitudinal analysis of PBMCs and serum from patients with breast cancer with AI therapy could identify diagnostic biomarkers, the relevant molecular pathways of disease pathogenesis, and potential therapeutic targets to correlate with murine samples from this AIIA model.

## 4. Materials and Methods

### 4.1. AIIA Induction and Sample Size Determination

BALB/C-Tg(NFκB-RE-luc)-Xen mice (1.5–6 mo of age) carrying a transgene containing six NFκB responsive elements and a modified firefly luciferase cDNA were purchased from Caliper Life Science (Hopkinton, MA, USA). The female NFκB-RE-luc mice were oophorectomized (N = 15) and, after a 4-week recovery period, were treated with an AI (letrozole; Sigma-Aldrich, St. Louis, MO, USA) via daily subcutaneous injections (10 µg/mouse/day) for 5 weeks. The control groups included oophorectomized mice (OVX) receiving vehicle (0.3% hydroxypropyl cellulose; HPC) injections (N = 10) and non-oophorectomized mice (sham surgery performed) treated with an AI (N = 15). An experimental summary of the AIIA animal model protocol is shown in Figure 1. Mouse maintenance and protocols were approved by the Institutional Animal Care and Use Committee at The Ohio State University Wexner Medical Center (OSUWMC). The animal facility was maintained at 22–23 °C and 30–50% relative humidity with a 12 h light/dark cycle; chow and water were supplied ad libitum. The sample sizes were based on calculations and data from previous studies, with a type one error of 0.05 and a power of 0.8 [9]. Specifically, the power calculation was performed with regard to the effect on letrozole-mediated treatment of status epilepticus. We anticipated that we would be able to reject the null hypothesis, as long as the true difference between the groups was 0.8 or greater on the probability scale.

### 4.2. Tissue Collection

Tissues were resected from each mouse and flash frozen in liquid-nitrogen-cooled isopentane, followed by cryosectioning, or they were immediately fixed by immersion in neutral-buffered 10% formalin and then processed into paraffin. Serial histological sections were stained with H&E (Leica Microsystems Inc., Buffalo Grove, IL, USA) following the manufacturer’s protocol, or they were labeled via immunohistochemistry (IHC) as detailed previously [30] and per the established protocols at HistoWiz (Brooklyn, NY). The histological slides were scanned for a downstream digital/quantitative image analysis.

### 4.3. Histopathology

The H&E-stained paraffin sections of tissue were subjected to a blinded histopathological analysis using the 10× objective of a bright-field light microscope with predefined criteria [30]. The scanned slide images were analyzed using Aperio ImageScope digital analysis software (v9.1) as detailed previously to determine positive staining and lymphocyte localization via immunohistochemistry [31]. Aperio’s positive pixel count algorithm was run to quantify the extent of positive staining and lymphocyte localization using calibrated hue, saturation, and intensity values following previously described methods of computer-assisted image analyses [31]. To define a mean positive pixel intensity value, 10 measurements of an identical total surface area of tissue were quantitated. All digital analyses were confirmed via manual slide interpretations using the 10× objective of the bright-field light microscope.

### 4.4. Cytokine Measurement

Serum was prepared from whole blood collected via the subclavian artery at the experimental endpoint. The serum samples were analyzed using an enzyme-linked immunosorbent assay (ELISA) with the V-PLEX Pro-inflammatory Panel 1 mouse kit (Meso Scale Diagnostics, Rockville, MD) according to the manufacture’s protocol, and the data were analyzed using Microsoft Excel (v2204). The limit of detection (LOD) of this kit for each analyte is as follows: IL-2—0.12 pg/mL, IL-6—0.12 pg/mL, IFN-γ—0.05 pg/mL, KC/GRO (CXCL1)—0.05 pg/mL, IL-4—0.04 pg/mL, and IL-5—0.07 pg/mL. All reported results were above the LOD of the assay.

### 4.5. Bioluminescent Imaging with IVIS

All IVIS imaging and analyses were performed as described previously [3]. In brief, the BALB/C-Tg(NFκB-RE-luc)-Xen mice were given 150 mg/kg luciferin (15 mg/mL in PBS pH 7; unadjusted) through intraperitoneal (IP) injections, and bioluminescent signals were captured using an IVIS 200 system (Caliper Life Sciences). Data were quantified and analyzed using IVIS Living Image^®^ software (v3.2).

### 4.6. MRI

The BioSpec 94/30 Imaging System with a 9.4 T horizontal bore magnet that operates at 400 MHz using ParaVision™ 5.1 software was used. MRI scans were acquired with a 72 mm volume coil (transceiver) with the following acquisition parameters: multi-spin multi-echo (MSME) method, echo time = 6.834 ms (minimum), 30 echos (6.834–136.68), repetition time = 2000 ms, averages = 2, resolution: 150*150 micron (FOV varied but resolution remained constant), and a slice thickness of 1 mm.

### 4.7. Letrozole Stimulation of Human PBMCs

PBMCs freshly isolated from healthy pre-menopausal females were cultured in hormone-free conditions as previously described [12] using RPMI 1640 (Life Technologies, Grand Island, NY) and 5% charcoal-stripped fetal bovine serum (FBS; Life Technologies). Participation was in accordance with an approved Institutional Review Board protocol at OSUWMC. The PBMCs were treated with 10 nM of 17β-estradiol (E2; Sigma) and/or 10 nM of letrozole (Sigma) and were collected at 36 hrs according to previously established protocols [12].

### 4.8. Statistics

A statistical analysis of cytokine detection and IVIS measurements for the three groups collectively was performed via one-way ANOVA. The digital pixilation of the IHC staining was analyzed via two-way ANOVA to estimate the mean quantitative variable changes according to the levels of both of the experimental groups (letrozole oophorectomy and letrozole sham) relative to each IHC stain. All ANOVA analyses were followed by Tukey’s post hoc test for multiple comparisons, and numerical datasets are expressed as mean values with standard error of the mean indicated (SEM) using Graph Pad Prism software (v8.3.0). Data were considered statistically significant if *p* ≤ 0.05.

## Data Availability

Data is contained within the article.
